# Doctors and Patients

**Published:** 1888-08-18

**Authors:** William Moore

**Affiliations:** K.C.I.E., late Surgeon-General with the Government of Bombay.


### Doctors and Patients.

By Sir William Moore, K.C.I.E., late Surgeon-General with the Government of Bombay.

Among the many reproaches which have been cast on medicine is the true one, that it is not an exact science. Perhaps the nearest approach to exactness in medicine, was the diagnosis and prescription of a last-century doctor who stated that a case consisted of want of power, cold, flabby, acid; and gave sal volatile to hold his patient up, ginger to warm him, and soda to correct him. But such "rule of thumb" prescriptions are not those of the present day. Although physicians may not yet have arrived at exactness, there is an intelligence in their giving of physic, which has first reduced the swallowing of nauseous drugs to a minimum, and has secondly divested medicine of the reproach cast upon it by Byron, who described it as "the destructive art of healing." It is too much the custom of the public to regard the human body as a machine, which when out of order may be set to rights by the doctors, much in the same manner as the watchmaker mends a watch. But if we consider how strange and wonderfully made is the human system, how complex are the functions it carries on for the preservation of life, and how liable it is to be affected by multitudinous causes, both external and internal, it will be at once seen that medicine can never arrive at the dignity of an exact science. There is a cause perhaps still more potent why it cannot do so; and this is the fact, that no two human beings are quite alike, either in features or in temperament, constitution, and habits. Some cynic observed that this difference from an assumed specific type is as a rule due to change towards a non-human form of existence. And he instanced gout in support of his statement, in which disease we have a mode of elimination in some essential particulars approaching to that normal in birds and reptiles. Our cynic also referred to certain skin affections, in which the structure of the skin is more or less transformed into that of a species of fish. Whether this is the case really or apparently, there are other reasons besides disease why all persons should not be alike. A person—to go no further back than his great grandfather—has some fourteen different hereditary sources, and it is well known that not only diseases, but temperaments, constitutions, and habits may be transmitted by heredity. Even the colour of the eyes is held to be transmitted in the proportions of one-fourth from parents and one-eighth from grandparents. It is also held that the hereditary transmission of any rare or valuable quality, or of any disease is checked by some opposing quality acquired from another source. We have the highest authority that the sins of fathers may be visited on the children. Even the habit of tobacco smoking in the parent may be the cause of weak heart in the children. The children of parents who have suffered from malarious fevers and malarious cachexia are, it is believed, more likely to acquire such maladies in after life. Even when no hereditary tendency can be traced, the idosyncrasies of human beings are numerous and remarkable. The distressing affection known as hay asthma must be attributed to some peculiar susceptibility of the sufferers to the pollen of hay floating in the atmosphere. Some persons are not, however, affected by hay, but get asthma, or spasmodic sneezing, or "colds in the head" from the aroma of peach blossoms, or of grape blossoms, or of Roman wormwood, or even of roses, and are sometimes so badly affected as almost to "die of a rose in aromatic pain." There are persons who cannot eat celery, shell fish, oatmeal cakes, or cucumber without suffering from nettlerash or colic, or perhaps from both ailments. Others cannot take sherry without suffering from acidity, and in some persons the slightest quantity of port wine excites gouty pains. The strong aversions some persons have to a cat, a spider, or an earwig, may also be regarded as an idiosyncrasy.

Perhaps, however, persons are oftener peculiarly susceptible to the action of medicines than to other agents. For instance, while the majority are acutely, and some few intensely, susceptible to the action of opium, there are persons who can take large quantities of the drug. It is the same with arsenic; people have been known to eat it like sugar, others suffer from irritation of the eyes and of the bowels after the smallest quantity. Ipecacuanha again affords an illustration. In some persons it immediately excites cough, sneezing, and watering of the eyes and nose. So strong is this susceptibility to ipecacuanha in some few persons, that they cannot go into a room in which there is even a sealed bottle of it. Quinine, in even the smallest quantity, such as quarter-grain doses, has been known to excite sore throat and eruptions on the skin. Santonine occasionally produces red-coloured urine, and green or yellow vision. Iodide of potassium frequently brings on sore throat and symptoms of "cold in the head." The smallest quantity of mercury will, in some cases, produce salivation. Very small doses of *dhatura strammonium* will, in some people, produce all the symptoms of poisoning by large doses in other persons. And so on might be added various other drugs, which act very differently on different people.

Now sex, habit, climate, temperament, constitution, race heredity, and idiosyncrasy, all exert influences which tend to render similar diseases in different people very varied in their characteristics; sometimes one class of symptoms being most prominent, sometimes another. And all this has to be considered by the prescribing physician. It is therefore the aim of the modern physician to treat each individual case in accordance with the peculiar symptoms and conditions presenting. It is thus evident that the popular idea of this medicine for that disease must be erroneous, and that patent medicines vaunted to cure all, or even many maladies, must be unequal to so desirable a result. It will now be understood why medicine cannot be the exact science we would wish it. It does not follow that what did one person good will be equally beneficial to others. Either sex, habit, climate, temperament, constitution, race, heredity, or idiosyncrasy may forbid it; or all may forbid it. Even the character of the prevailing disease may forbid it; and this often occurs during epidemics, when the treatment found efficacious in former epidemics is found useless on a recurrence. This has often been noticed, especially in cholera epidemics for example. But the public do not sufficiently understand that, as in other matters, so in medicine, there may be two or more ways or methods of arriving at the same place or position, or of achieving a desired object. One physician may treat his patients in one way, another physician may treat his patients in a different manner. If one is successful and the other is not, the latter is often unduly blamed, whereas the obstacle to success may be in some ill-defined peculiarity of the patient's temperament, constitution, or idiosyncrasies. Again, many questions asked by physicians are sometimes regarded as unnecessary or prying. But it is necessary for the medical man to know all about his patient before he can suitably, or even safely prescribe. In Luther's memoirs it is stated that the lame, halt, blind, deaf, dumb, and diseased, are possessed by devils, and that physicians who pretend to treat such infirmities as resulting from natural causes, are mere quacks, and totally ignorant of devils. We have happily advanced since Luther's time, and do not attribute diseases to devils or epidemics to the anger of Providence. We attribute diseases to causes which are daily becoming more and more known, and we rightly attribute epidemics of most kinds to filth and want of sanitation. In such matters the public are mostly in accord with their doctors. But still greater confidence is required. The medical profession have abandoned the *omne ignotum pro magnifico* which formerly invested them. Many doctors even no longer write in dog Latin. In return the sick public, if they desire care or relief, must place still great confidence in their doctors; they must make their doctors acquainted with everything concerning them, considering nothing too trivial to impart. For the doctor, when he prescribes for his patients, has, as previously shown, a multitude of considerations passing through his mind. And it is by such considerations that he arrives at the diagnosis of his case and the line of treatment proper to adopt. But his patient must use his best endeavours to supply the means.

